# Single and Combined Effects of Polystyrene Nanoplastics and Dibutyl Phthalate on Hybrid Snakehead (*Channa maculata* ♀ × *Channa argus* ♂)

**DOI:** 10.3390/antiox14091084

**Published:** 2025-09-03

**Authors:** Mi Ou, Ziwen Yang, Yuntao Lu, Yang Zhang, Yang Zou, Yueying Deng, Yuandong Sun, Haiyang Liu, Qing Luo, Shuzhan Fei, Kunci Chen, Dandan Gao, Jian Zhao

**Affiliations:** 1 Key Laboratory of Tropical and Subtropical Fishery Resources Application and Cultivation, Ministry of Agriculture and Rural Affairs, Pearl River Fisheries Research Institute, Chinese Academy of Fishery Sciences, Guangzhou 510380, China; om1990@prfri.ac.cn (M.O.); 17707276905@163.com (Z.Y.); 17633537502@163.com (Y.L.); dy211004@163.com (Y.Z.); zy20030430zy@163.com (Y.Z.); dyy12504@163.com (Y.D.); hyliu@prfri.ac.cn (H.L.); luoqing@prfri.ac.cn (Q.L.); feisz@prfri.ac.cn (S.F.); chenkunci@prfri.ac.cn (K.C.); 2 School of Fishery, Zhejiang Ocean University, Zhoushan 316022, China; 3 School of Life and Health Sciences, Hunan University of Science and Technology, Xiangtan 411201, China; syd@hnust.edu.cn; 4 Suqian Institute of Agricultural Sciences, Jiangsu Academy of Agricultural Sciences, Suqian 223800, China; 5 College of Fisheries and Life Sciences, Shanghai Ocean University, Shanghai 201306, China

**Keywords:** aquaculture, histopathology, antioxidant capacity, qPCR, intestinal microbiota, PSNPs, DBP

## Abstract

The ecological impact of microplastic pollution in freshwater ecosystems has received growing scientific attention, although research on freshwater species remains limited compared to marine organisms. This study investigates the individual and combined toxicological impacts of polystyrene nanoparticles (PSNPs) and dibutyl phthalate (DBP) on hybrid snakehead (*Channa maculata* ♀ × *Channa argus* ♂), a commercially important freshwater fish. PSNPs inhibited growth, induced hepatic and intestinal lesions, and delayed ovarian development, co-exposure with DBP exacerbated these effects. qPCR analysis revealed significant up-regulation of inflammation-related genes in the liver but inhibitory effects in the intestines, indicating that PSNPs and DBP provoke immune modulation and systemic pro-inflammatory responses. Furthermore, PSNPs and DBP induced oxidative damage in the liver and intestines by affecting antioxidant enzyme activity. 16S rRNA sequencing revealed that PSNPs and DBP altered intestinal microbiota composition, particularly reducing Proteobacteria abundance. Correlation analyses indicated negative associations between the abundances of Proteobacteria and Firmicutes and antioxidant parameters (SOD and MDA), suggesting microbiota-mediated impacts on host metabolism and physiological health. These findings highlight the ecological threat of microplastics and phthalates in freshwater environments and underscore the need for targeted conservation strategies.

## 1. Introduction

Micro/nanoplastics (M/NPs) are emerging pollutants that infiltrate aquatic ecosystems through wastewater discharge, landfill leakage, agricultural irrigation, and industrial emissions, establishing these environments as major M/NP reservoirs [1,2]. They have been detected globally across multiple environmental matrices, including the atmosphere, aquatic systems (including marine and freshwater environments), and terrestrial and sedimentary systems [3].These plastic particles can pose a certain degree of biological risk, including growth inhibition, histopathological damage, and metabolic disruption, such effects may persist transgenerationally via reproductive impairment [4,5,6]. Notably, some freshwater ecosystems often exhibit M/NP concentrations comparable to marine systems, with riverine discharge constituting a primary source of marine microplastic pollution [7], potentially exacerbating ecological risks in freshwater habitats. Several field studies have reported the presence of microplastics in freshwater habitats and in the gastrointestinal tracts of wild freshwater fish [8,9]. This issue has raised widespread concern about the potential impacts of microplastics on ecosystems and human health through the food chain [3]. Due to their hydrophobicity and high surface area, M/NPs readily adsorb organic pollutants, altering the physicochemical properties and ecotoxicity of both materials; however, their combined effects remain poorly understood. While M/NPs typically induce broad-spectrum toxicity (e.g., oxidative stress, inflammation, metabolic disruption), organic pollutants often target specific organs (e.g., liver, thyroid, reproductive tissues) or cellular processes. Interactions during co-exposure may be synergistic or antagonistic [10]. These divergent outcomes depend on M/NP properties—polymer type, surface charge, and hydrophobicity—which govern pollutant binding and bioavailability. Moreover, additives like plasticizers further influence M/NP behavior and toxicity [11].

DBP, a common plasticizer used to enhance polymer flexibility, leaches readily from plastics due to non-chemical bonding. DBP exposure has been linked to endocrine disruption in aquatic organisms, impairing reproductive behaviors and fertility [12]. Chronic DBP exposure also disrupts antioxidant enzyme activity (e.g., superoxide dismutase, glutathione peroxidase), compromising oxidative stress resistance [13]. Given the widespread presence of M/NPs and DBP in freshwater ecosystems, fish inevitably face prolonged ingestion or exposure to these contaminants.

The hybrid snakehead (*Channa maculata* ♀ × *Channa argus* ♂) is a commercially important freshwater fish in China, valued for its rapid growth, high nutritional value, less intermuscular spines, and medicinal properties [14]. Its benthic and midwater habitat increases MPs ingestion risk due to pollutant accumulation in these zones. To evaluate individual and combined toxicities of nanoplastics and plasticizers, we assessed impacts on the hybrid snakehead for growth, oxidative stress, inflammatory responses, and gut microbiota to elucidate toxicity mechanisms of these pollutants. These findings are expected to provide preliminary insights into the toxicological effects of emerging pollutants and hold significant importance for the healthy farming of hybrid snakeheads.

## 2. Materials and Methods

### 2.1. Chemical Reagents

Fluorescent PSNPs (80 nm, 10 mg/mL) were purchased from Bessler Chromatography Technology Development Center (Tianjin, China). The specific characterization data can be referred to in the study by Wang et al. [15]. DBP was obtained from Aladdin Reagents (Shanghai, China). Assay kits for total protein (TP, A045-2-2), superoxide dismutase (SOD, A001-3), catalase (CAT, A007-1), and malondialdehyde (MDA, A003-1) were obtained from Nanjing Jiancheng Bioengineering Institute (Nanjing, China).

### 2.2. Experimental Fish

Female hybrid snakeheads were obtained from Guangdong Bairong Aquatic Quality Breeding Group Co., Ltd. (Foshan, China) to eliminate potential sex-related variability. Fish were acclimated for two weeks in a recirculating aquaculture system at the Pearl River Fisheries Research Institute, Chinese Academy of Fishery Sciences, under controlled conditions: 15:9 light-dark cycle, temperature 27.5–30.0 °C, pH 6.5–7.5, dissolved oxygen 5.0–7.0 mg/L (with continuous aeration), and ammonia nitrogen <0.1 mg/L. Commercial feed was provided during acclimation, followed by a 24 h fast before experimentation.

### 2.3. Experimental Procedure

PSNPs exposure: feed (150 g/group) was immersed in 100 mL ammonia-free water and coated with green fluorescent PSNPs at concentration of 0 (PSNPs-Ctrl), 20 (PSNPs-Low), 100 (PSNPs-Medium), or 500 mg/kg (PSNPs-High), following modified methodologies from Lai et al. [16] and Junaid et al. [17]. After hydration-induced expansion, feed was stirred (5 min), oven-dried (37 °C, 24 h), and stored at 4 °C in darkness. Three hundred size-matched female fish, 2 months old (length: 10.00 ± 0.25 cm; weight: 12.50 ± 0.50 g) were randomly distributed into four treatment groups (75 fish/group), with triplicate 165 L tanks per group (25 fish/tank, 12 tanks total). Fish were fed twice daily (09:00 and 17:00) for 21 days under continuous aeration.

**Figure 1:**
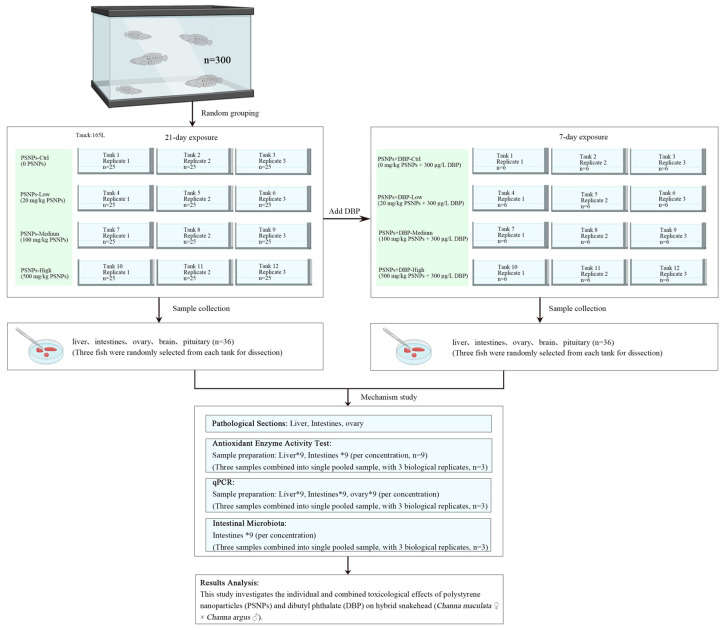
Experimental flowchart illustrating the toxicological effects of PSNPs and DBP on hybrid snakehead.

Co-exposure with DBP: after initial sampling, remaining fish underwent 2 days acclimatization in clean water. Eighteen fish per group were transferred to 12 new tanks (6 fish/tank) and exposed to 300 μg/L DBP; lowest observed effect concentration reported for zebrafish embryos [18]). DBP was dissolved in Dimethyl sulfoxide (DMSO; 10 mL), diluted 1:9 (*v*/*v*) with deionized water, homogenized, and added to tanks. Four co-exposure groups were designated: PSNPs+DBP-Ctrl (0 mg/kg PSNPs + 300 μg/L DBP), PSNPs+DBP-Low (20 mg/kg PSNPs + 300 μg/L DBP), PSNPs+DBP-Medium (100 mg/kg PSNPs + 300 μg/L DBP), and PSNPs+DBP-High (500 mg/kg PSNPs + 300 μg/L DBP). Sampling followed 7 days of co-exposure (Figure 1).

It is noteworthy that the dose gradient in this study spans from environmentally relevant to extreme concentrations. Typical environmental levels of PSNPs in freshwater sediments range from a few to several tens of mg/kg [19,20,21], with reported values including an average of 11.80 mg/kg in stormwater ponds [19] and a high of 104.0 mg/kg in the Brisbane River [20]. In severely contaminated areas like road runoff zones, concentrations can reach tens of thousands of mg/kg [21]. Accordingly, the low dose used in this study (20 mg/kg) represents the upper limit of common environmental exposure, while the medium (100 mg/kg) and high (500 mg/kg) doses simulate severe pollution scenarios to assess potential biological effects. Similarly, phthalate esters (e.g., DBP and DEHP) also exhibit considerable variability in environmental concentrations. DEHP levels reported in freshwater ecosystems range from 98 to 13,050 μg/L, with levels as low as 100–800 μg/L shown to cause significant reductions in cell viability and induce DNA damage [17]. DBP, a homologous compound of DEHP, is more commonly detected in aquaculture areas and presents a non-negligible ecological risk. Therefore, DBP was set at 300 μg/L, a concentration within the reported effect range but not overly toxic, making it suitable for examining dose–response relationships and mechanistic endpoints.

### 2.4. Sample Collecting

Fish were fasted for 24 h, anesthetized, and sampled post-exposure. Three individuals per tank were dissected, the liver, intestines, ovary, brain, and pituitary were collected. Tissues of the same type from three fish were pooled to create one composite sample, resulting in three biological replicates per experimental group. Tissues for biochemical assays (antioxidants, gene expression, microbiome) were stored at −80 °C. Liver, intestine, and ovary samples for histology were fixed in 4% paraformaldehyde (PFA). Ovarian, hepatic, and intestinal tissues were histologically assessed via hematoxylin-eosin (H&E) staining [22]. Additionally, to comprehensively evaluate the physiological effects of PSNP exposure, morphometric parameters (body length and weight) were recorded at the end of the exposure experiment, and growth indices were calculated according to a standard procedure [23].

### 2.5. Quantitative Real-Time PCR (qPCR)

Total RNA was extracted from ovary, liver, intestines, and pituitary using TRIzol reagent (Invitrogen, Carlsbad, CA, USA). RNA integrity was assessed via 1% agarose gel electrophoresis, and concentrations were measured using a BioSpectrometer (Thermo Fisher, Dreieich Germany). First-strand cDNA synthesis employed the PrimeScript RT kit (TaKaRa, Otsu, Japan), with products stored at −20 °C.

Primers (Table S1) were designed against the *Channa maculata* genome (SRA Accession No. PRJNA730430) using Primer Premier 5.0 (Premier Biosoft, Palo Alto, CA, USA). Target genes included seven Inflammation markers (*IL-8*, *IL-1β*, *IL-10*, *TOR*, *IκBα*, *NF-κB*, *SOD*), five growth regulators (*GHR*, *IGF1-1*, *IGF1-2*, *IGF2*, *GH*), and fifteen gonadal factors (*Bmp15*, *Ctnd1*, *Ctnd2*, *Cyp19a1a*, *Er*, *Figla*, *Foxl2*, *Pax4*, *Sox11b*, *Sox3*, *Amh*, *Amhr2*, *Dmrt1*, *Star*, *Sox11a*). qPCR was performed in triplicate on a StepOnePlus Real-Time PCR System (Applied Biosystems, Foster City, CA, USA) with *β-actin* as the reference gene. The 2^−ΔΔCt^ method was applied to normalize the Ct values of each reaction, with the control group’s expression level assigned a value of 1.0.

### 2.6. Biochemical Analysis

Liver and intestinal oxidative stress markers (MDA, SOD, CAT) were quantified using commercial kits (Nanjing Jiancheng, China). Tissues were homogenized in 0.9% saline to prepare 10% (*w*/*v*) homogenates, centrifuged (2500 rpm, 10 min, 4 °C), and supernatants were collected for analysis. Total protein (TP) was measured via the Coomassie Brilliant Blue method, and oxidative stress markers were normalized to TP concentrations. The tissue samples were divided into three groups with three replicates each. Enzyme activity in all concentration groups was measured with nine independent replicates.

### 2.7. Intestinal Microbiota Analysis

For each treatment group, intestinal tissues from nine fish were collected and pooled in sets of three to generate one composite sample, yielding three biological replicates. The 16S rRNA V3–V4 region was amplified using primers 338F (5′-ACTCCTACGGGAGGCAGCAG-3′) and 806R (5′-GGACTACHVGGGTWTCTAAT-3′), purified with the Omega DNA Purification Kit (Omega, Norcross, GA, USA), and sequenced on the Illumina NovaSeq 6000 platform (2 × 250 bp paired-end) by Biomarker Biotechnology Co., Ltd. (Shanghai, China). Raw sequencing reads were first quality-filtered using Trimmomatic v0.33, followed by primer detection and removal with cutadapt v1.9.1, resulting in primer-free clean reads. Denoising was then performed with the DADA2 pipeline implemented in QIIME2 v2020.6 [24,25], during which paired-end reads were merged and chimeric sequences removed, producing high-quality sequences.

Sequences with ≥97% similarity were clustered into operational taxonomic units (OTUs) using USEARCH v10.0. Taxonomic classification was carried out with the Naive Bayesian classifier in QIIME2 against the SILVA v138.1 database, applying a 70% confidence threshold. Alpha diversity indices (Chao1, Shannon, Simpson, and ACE) were calculated with QIIME2 to evaluate microbial richness and diversity, while beta diversity was assessed using principal coordinate analysis (PCoA) to compare community composition across groups. Differences in bacterial abundance were statistically tested using one-way analysis of variance (ANOVA).

### 2.8. Data Analysis

Data are presented as mean ± standard error of the mean (SEM) unless otherwise indicated. Statistical analyses were performed using one-way analysis of variance (ANOVA) with Duncan’s post hoc test, employing SPSS 25.0 software (IBM, Armonk, NY, USA). Statistical significance was defined as *p* < 0.05.

## 3. Results

### 3.1. Growth Assessment

After 21 days of PSNPs exposure, survival rates (SR) showed no significant differences among groups (*p* > 0.05). However, final body weight (FBW), weight gain rate (WGR), and specific growth rate (SGR) were significantly reduced in the PSNPs-Medium and PSNPs-High groups (*p* < 0.05) (Table 1), with SGR decreasing by 10.20% and 19.60%, respectively. These results suggest dose-dependent growth inhibition from dietary PSNPs exposure.

### 3.2. Histopathological Analysis

**Figure 2:**
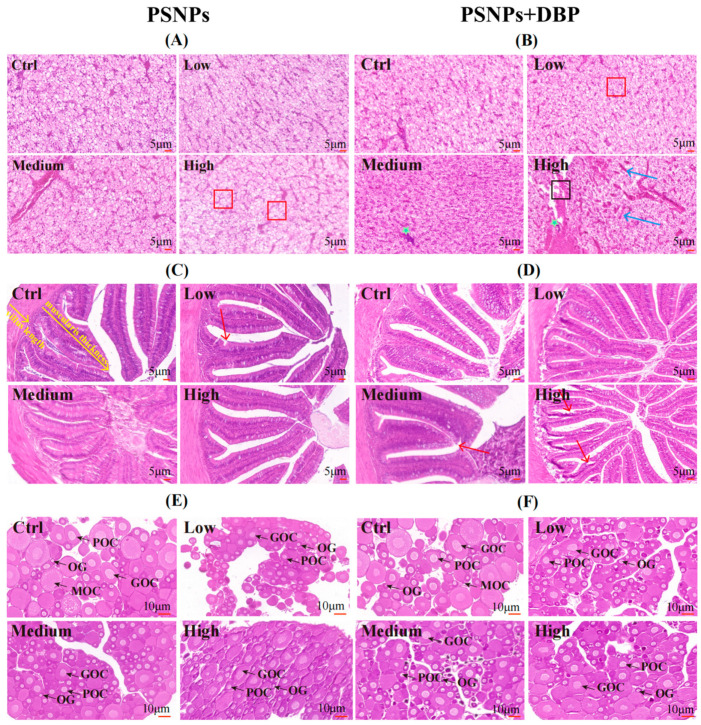
Histopathological alterations in the liver, intestines, and ovary of hybrid snakeheads following exposure to PSNPs alone (**A**,**C**,**E**) or in combination with DBP (**B**,**D**,**F**). The red box indicates hepatocyte swelling and cytoplasmic vacuolation, the black box highlights liver tissue damage, the green asterisk marks inflammatory cell infiltration, the blue arrow points to sinusoidal dilation, and the red arrow indicates intestinal villi wear. OG, oogonia; POC, primary oocyte; GOC, growing oocyte; MOC, mature oocyte.

High-dose PSNPs induced hepatocytes vacuolation (Figure 2A-High), and co-exposure exacerbated damage, causing diffuse vacuolation (Figure 2B-Low), inflammatory infiltration (Figure 2B-Medium), and sinusoidal dilation with structural disruption (Figure 2B-High). Intestinal sections showed villous epithelial erosion in the PSNPs-Low group (Figure 2C-Low), progressing to atrophy and epithelial sloughing under co-exposure (Figure 2D-Medium, Figure 2D-High). Statistical analysis of damaged villi revealed that after PSNPs alone exposure, the villus damage rate in the PSNPs-Low group was higher, and the villus damage rate after co-exposure with DBP was higher than that of the PSNPs alone exposure group (Table 2). Controls exhibited intact follicular stages, including oogonia (OG), primary oocytes (POC), growing oocytes (GOC), and mature oocytes (MOC) (Figure 2E-Ctrl, Figure 2F-Ctrl). Exposed groups showed arrested folliculogenesis, with increased OG/POC and reduced/absent MOC.

### 3.3. Gene Expression Analyses

#### 3.3.1. Growth-Related Genes

**Figure 3:**
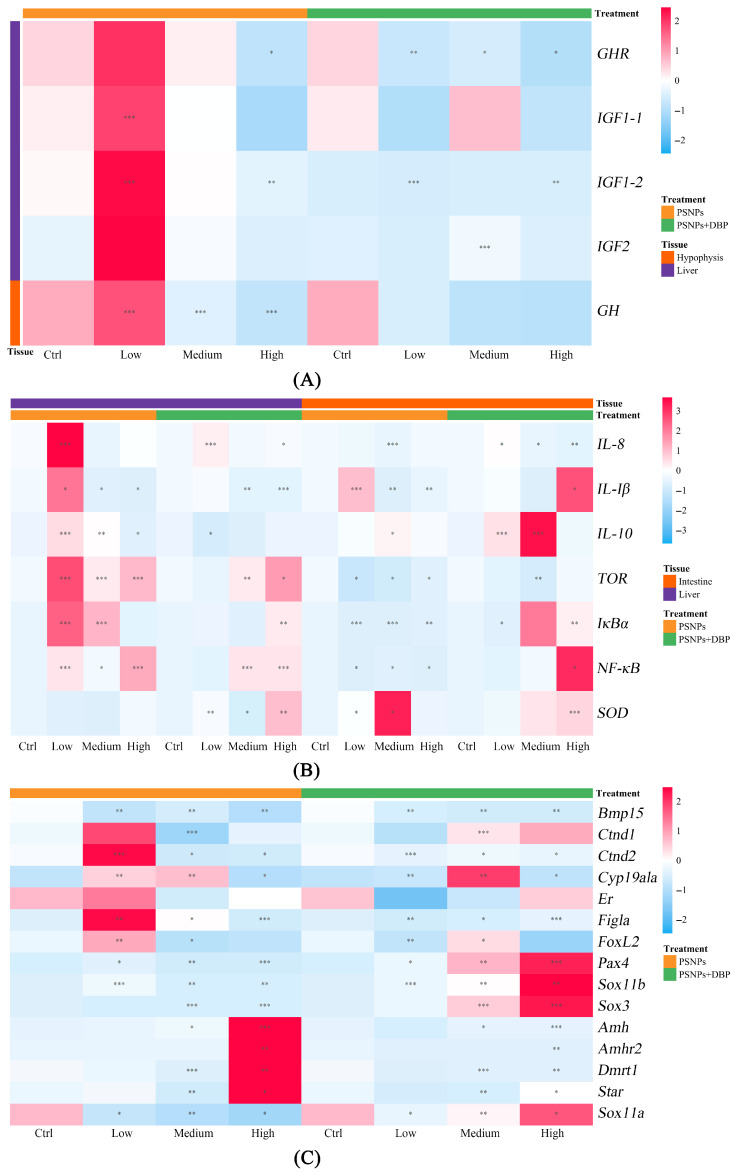
mRNA expression profiles of growth-related (**A**), inflammation-related (**B**), and gonadal-related genes (**C**) in hybrid snakehead after exposure to PSNPs alone or in combination with DBP. * denotes statistically significant difference (*p* < 0.05) versus the corresponding control group (PSNPs-Ctrl or PSNPs+DBP-Ctrl), ** represents high significant difference (*p* < 0.01), and *** indicates extremely significant difference (*p* < 0.001).

Low-dose PSNPs significantly up-regulated *GH, IGF1-1*, and *IGF1-2* (*p* < 0.05), while higher doses led to dose-dependent down-regulation of *GHR, IGF1-2*, and *GH* (*p* < 0.05). Co-exposure with DBP further suppressed GH/IGF axis genes compared to PSNPs alone (Figure 3A).

#### 3.3.2. Inflammation-Related Genes

To evaluate immunological responses, liver and intestinal expression of inflammation-related genes was assessed. In the liver, PSNPs induced a biphasic response (Figure 3B): significant up-regulation of all cytokines in the PSNPs-Low group (*p* < 0.05), partial elevation (*TOR*, *IκBα*, *NF-κB*) in the PSNPs-Medium group, and sustained expression of *TOR* and *NF-κB* in the PSNPs-High group. In the intestines, overall inflammatory gene expression decreased, though specific genes showed dose-dependent up-regulation. Notably, *IL-1β* mRNA levels were significantly increased in the PSNPs-Low group, *IL-10* in the PSNPs-Medium group, and *SOD* in both the PSNPs-Low and PSNPs-medium groups.

Compared to PSNPs-only exposure, the low-dose co-exposure group showed a reduction in hepatic inflammatory gene expression. However, in the high-dose co-exposure group, the relative expression levels of *TOR, IκBα, NF-κB,* and *SOD* in the liver were significantly increased, along with notable up-regulation of *IL-1β, IκBα, NF-κB*, and *SOD* in the intestines in the high-dose co-exposure group.

#### 3.3.3. Gonadal-Related Genes

The expression profiles of genes associated with gonadal function (*Bmp15, Ctnd1, Ctnd2, Cyp19a1a, Er, Figla, FoxL2, Pax4, Sox11b, Sox3, Amh, Amhr2, Dmrt1, Star, Sox11a*) were evaluated (Figure 3C). The study found that in the PSNPs-Medium and PSNPs-High exposure groups, the expression levels of *Ctnd1, Pax4, Sox11b, Sox3,* and *Sox11a* were significantly down-regulated, while *Amh, Amhr2, Dmrt1,* and *Star* were significantly up-regulated only in the PSNPs-High group and suppressed in the PSNPs-Low and PSNPs-Medium groups. Upon co-exposure with DBP, the mRNA levels of *Ctnd1, Pax4, Sox11b, Sox3*, and *Sox11a* were up-regulated in both the PSNPs+DBP-Medium and PSNPs+DBP-High groups, while the expression of *Amh, Amhr2*, and *Dmrt1* was significantly suppressed in the same groups. Notably, *Bmp15, Ctnd2, Cyp19a1a, Figla,* and *Foxl2* were consistently down-regulated in both the PSNPs-High and PSNPs+DBP-High exposure groups.

### 3.4. Antioxidant Enzyme Activity Analysis

**Figure 4:**
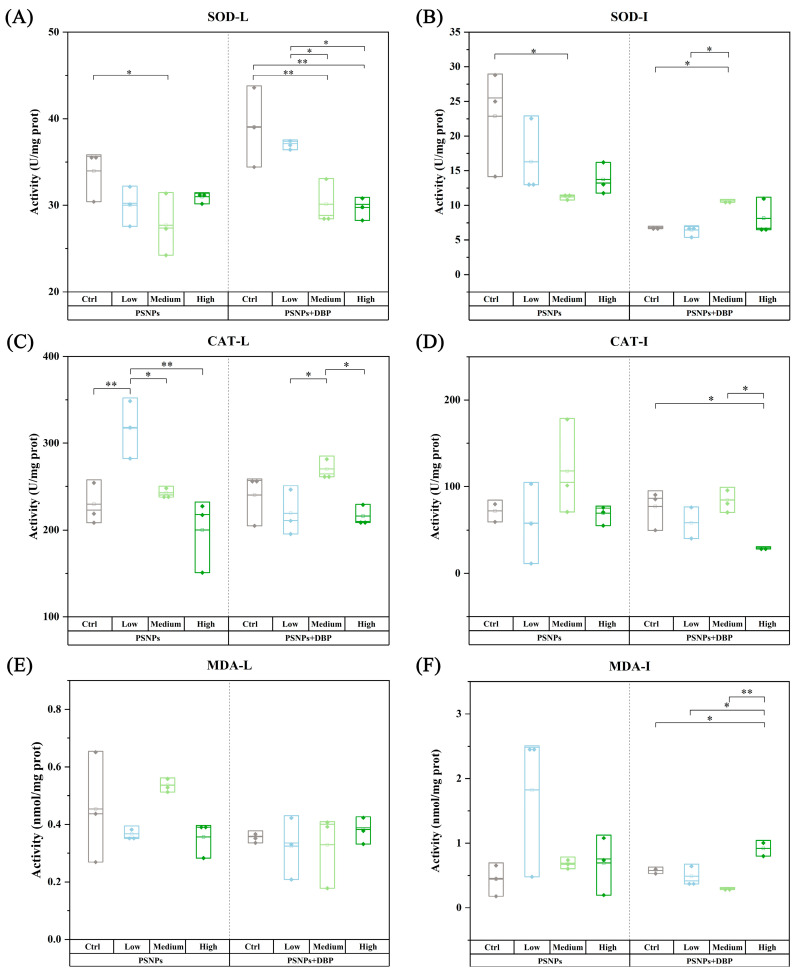
Enzymatic activity profiles of SOD, CAT, and MDA in liver (L) and intestines (I) of hybrid snakehead following exposure to PSNPs alone or in combination with DBP. (**A**,**B**) SOD activity in liver and intestines; (**C**,**D**) CAT activity in liver and intestines; (**E**,**F**) MDA levels in liver and intestines. The central line within the box denotes the median, while the box boundaries define the interquartile range (25th to 75th percentiles). SOD, Superoxide dismutase; CAT, Catalase; MDA, Malondialdehyde. * denotes statistically significant difference (*p* < 0.05) versus the corresponding control group (PSNPs-Ctrl or PSNPs+DBP-Ctrl), ** represents high significant difference (*p* < 0.01).

Liver (L) and intestines (I) antioxidant responses under PSNPs and PSNPs+DBP exposure are shown in Figure 4. In the PSNPs-only groups, hepatic and intestinal SOD activity significantly declined in the medium-dose group (*p* < 0.05), while hepatic CAT activity increased in the low-dose group. Hepatic MDA, intestinal CAT, or intestinal MDA levels remained unchanged (*p* > 0.05). Under co-exposure, hepatic SOD showed a dose-dependent decrease, whereas intestinal SOD increased in the medium group (*p* < 0.05). Hepatic CAT remained largely unchanged, though medium-dose co-exposure elevated its activity relative to low and high doses. Intestinal CAT was significantly reduced in the high-dose co-exposure group (*p* < 0.05). While hepatic MDA remained stable, intestinal MDA significantly increased in the high-dose co-exposure group. These results indicate that PSNPs impair antioxidant defenses, and DBP co-exposure further exacerbates oxidative stress in a tissue-specific manner.

### 3.5. Intestinal Microbiota Composition

The rarefaction curve results showed that with the increase in sequencing reads, species accumulation curves for all treatment groups rose rapidly at first and then gradually leveled off, indicating that the sequencing depth was sufficient to cover most of the microbial diversity within the samples (Figure S1). In addition, coverage indices for all samples were close to 1.0 (Table 3), further confirming that the sequencing depth was adequate for subsequent diversity analyses. PSNPs-only exposure reduced ACE and Chao1 indices relative to controls, while co-exposure with DBP slightly increased them; however, neither change was significant (*p* > 0.05). Similarly, Shannon and Simpson indices remained stable, indicating that neither PSNPs nor PSNPs+DBP significantly altered intestinal microbiota diversity under these experimental conditions.

**Table 3:** Diversity indices of the hybrid snakehead gut microbiota at the OTU level.

Group	Chao1	Simpson	Shannon	ACE	Coverage
PSNPs-Ctrl	783.34	0.99	8.07	783.80	1
PSNPs-Low	765.25	0.97	6.53	767.79	0.999
PSNPs-Medium	757.45	0.96	6.98	759.31	0.999
PSNPs-High	745.70	0.99	8.16	746.86	0.999
PSNPs+DBP-Ctrl	840.03	0.98	7.13	841.11	0.999
PSNPs+DBP-Low	918.34	0.98	7.86	918.87	1
PSNPs+DBP-Medium	885.39	0.98	7.28	886.48	0.999
PSNPs+DBP-High	893.03	0.96	7.24	894.03	0.999

**Figure 5:**
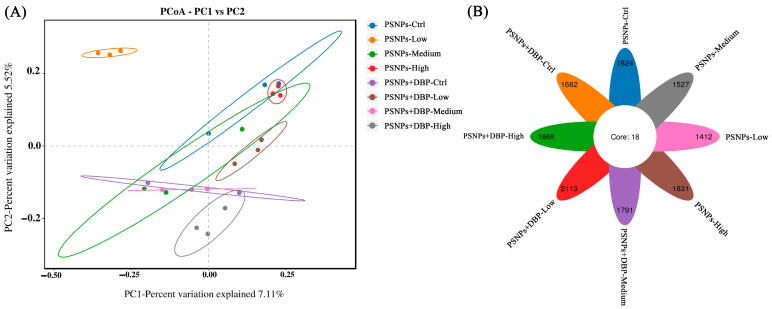
PCoA analysis (**A**) and Venn diagram (**B**) of the gut microbiota composition in hybrid snakehead.

β-diversity analysis, assessed via unweighted UniFrac-based PCoA, revealed distinct clustering of the PSNPs-High group microbiota compared to other treatments, though intra-group variability was pronounced in the PSNPs-Low and PSNPs+DBP-Ctrl groups (*p* > 0.05; Figure 5A). The Venn diagram identified 18 OTUs were shared across all groups. Group-specific OTUs numbered 1824 in PSNPs-Ctrl, 1412 in PSNPs-Low, 1527 in PSNPs-Medium, 1831 in PSNPs-High, 1682 in PSNPs+DBP-Ctrl, 2113 in PSNPs+DBP-Low, 1791 in PSNPs+DBP-Medium, and 1868 in PSNPs+DBP-High group (Figure 5B).

**Figure 6:**
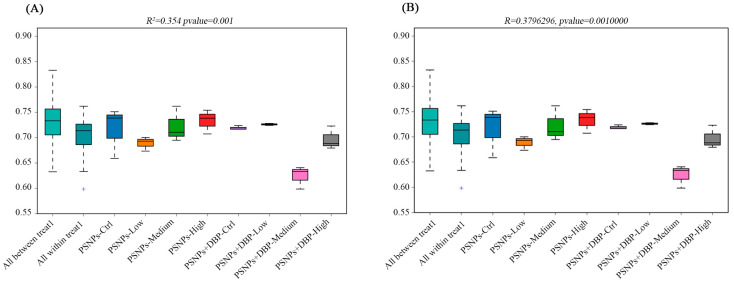
β-diversity analysis of gut microbiota based on unweighted UniFrac distances. (**A**) PERMANOVA showed that treatment explained 35.4% of the variation (R^2^ = 0.354, *p* = 0.001); (**B**) ANOSIM also indicated significant differences among groups (R = 0.380, *p* = 0.001).

In addition, β-diversity indicated that gut microbial community structures differed to some extent among the treatment groups. PERMANOVA analysis showed that treatment explained approximately 35.4% of the variation (R^2^ = 0.354, *p* = 0.001) (Figure 6A), suggesting that treatments may have influenced community composition. Consistently, ANOSIM analysis supported this trend (R = 0.380, *p* = 0.001) (Figure 6B). Further comparisons revealed that the PSNPs+DBP-Low and PSNPs+DBP-Ctrl groups exhibited relatively homogeneous community structures, whereas the PSNPs+DBP-Medium group and high-dose groups (e.g., PSNPs-High) showed greater variability. Overall, these findings suggest that PSNPs exposure and co-exposure with DBP may affect intestinal microbial community structures, although the extent of these effects and the underlying mechanisms require further investigation.

**Figure 7:**
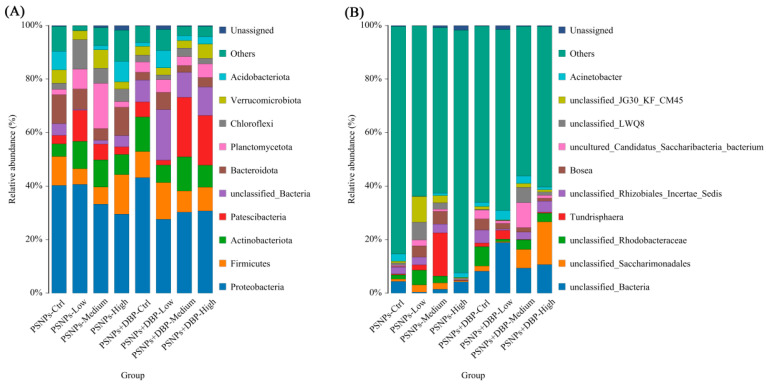
Gut microbiota composition in hybrid snakeheads at phylum (**A**) and genus (**B**) levels following exposure to PSNPs alone or in combination with DBP.

At phylum level, dominant gut microbiota included Bacteroidota, Proteobacteria, Actinobacteriota, Firmicutes, Chloroflexi, and Planctomycetota across all groups (Figure 7A). Proteobacteria was most abundant, exceeding 40% in the PSNPs-Ctrl, PSNPs-Low, and PSNPs+DBP-Low groups. At genus level, *Acinetobacter*, *Bosea*, and *Tundrisphaera* predominated (Figure 5B), with *Acinetobacter* markedly enriched in co-exposure groups compared to PSNPs-only groups.

### 3.6. Correlation Analysis Among Gut Microbiota, Inflammatory Genes, and Antioxidants Indices

**Figure 8:**
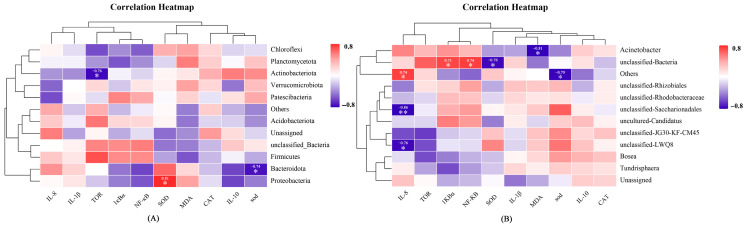
Spearman correlation analysis between the gut microbiome, inflammatory genes, and antioxidant markers. (**A**) The relationship between gut microbiota at the phylum level and inflammatory genes/antioxidant indicators. (**B**) The relationship between gut microbiota at the genus level and inflammatory genes/antioxidant indicators. * indicates statistical significance (*p* < 0.05); ** indicates high statistical significance (*p* < 0.01).

Spearman correlation analysis was employed to evaluate relationships among gut microbiota abundance (phylum or genus levels) and the expression of inflammatory genes, as well as antioxidant markers in hybrid snakeheads (Figure 8). Proteobacteria abundance positively correlated with SOD (r = 0.81, *p* < 0.05), while Bacteroidota and Actinobacteriota showed negative correlations with SOD (r = −0.74, *p* < 0.05) and TOR gene expression *(*r = −0.76, *p* < 0.05), respectively. At the genus level, *Acinetobacter* was negatively correlated with MDA (r = −0.81, *p* < 0.05). These correlations suggest potential microbiota-mediated regulation of redox and immune responses.

## 4. Discussion

Growth suppression is a key endpoint in evaluating the ecotoxicity of PSNPs and their interactions with organic pollutants. Most studies indicate microplastic-associated growth impairment in fish [26,27], although some show neutral responses [28], likely due to variations in particle properties, exposure routes, durations, or species-specific sensitivity. Nanoplastics cause developmental inhibition in *D. rerio* [29], and PVC exposure induces somatic growth retardation in *Cyprinus carpio* 22[]. In this study, hybrid snakeheads exhibited a biphasic response to PSNPs, moderate weight gain at low-dose but significant growth reduction at higher concentrations, suggesting hormesis followed by toxicity. The growth inhibition may result from elevated metabolic costs of detoxification and inflammation, coupled with digestive lesions and nutrient malabsorption-induced pseudosatiety [30]. Although PSNPs exposure did not affect survival rates, the observed growth deficits imply sublethal physiological stress. This implies that current environmental M/NPs levels may impair nutrient assimilation and energy allocation without causing acute mortality.

To further investigate the mechanisms underlying PSNP-induced growth effects, we analyzed gene expression in the growth hormone/insulin-like growth factor (GH/IGF) axis, a conserved endocrine pathway regulating fish growth. Low-dose PSNPs up-regulated *GH, GHR, IGF1-1, IGF1-2,* and *IGF2* mRNA in the liver and pituitary, potentially reflecting a compensatory activation associated with early growth stress [31,32]. In contrast, co-exposure with DBP significantly down-regulated these genes, which may be indicative of an aggravated disruption of the GH/IGF axis. This pattern is consistent with a potentially weakened compensatory endocrine response to early growth stress and further affect growth.

Histopathological analysis revealed PSNP-induced liver and intestinal damage, exacerbated by DBP co-exposure. Although PSNPs are insoluble, their nanoscale size enables translocation across intestinal barriers and hepatic accumulation via systemic circulation. The lipophilic endocrine disruptor DBP readily crosses cell membranes, binding nuclear receptors (e.g., PPARs) and disrupting lipid metabolism and oxidative balance. PSNPs act as pollutant carriers, enhancing DBP uptake and tissue distribution. Therefore, we speculate that combined exposure to PSNPs and DBP may be associated with more severe hepatic and intestinal tissue damage. Both PSNPs alone and in combination with DBP significantly reduced mature oocyte proportions, indicating impaired ovarian development, which is consistent with gonadal suppression induced by N/MPs reported in other freshwater teleosts [33,34,35]. Mechanistically, PSNPs may disrupt hypothalamic-pituitary-gonadal (HPG) axis signaling and induce oxidative ovarian injury. Co-exposure likely enhances endocrine disruption through elevated DBP accumulation in ovarian tissue, contributing to delayed folliculogenesis and potential early ovarian dysfunction. Although practical constraints, including facility capacity and the multi-omics sample processing complexity, precluded a fully factorial design to elucidate dose–response relationships, this study provides preliminary evidence that co-exposure at environmentally relevant doses exacerbates tissue pathology that PNSPs induce histopathological damage in hybrid snakehead, with co-exposure to DBP exacerbating this effect. This finding establishes a methodological foundation for future dose–response and interaction analysis.

Oxidative stress, characterized by lipid peroxidation and suppression of antioxidant enzymes, is a well-documented toxicological effect of microplastic exposure in aquatic organisms [36,37]. In hybrid snakehead, both PSNPs alone and co-exposure with DBP significantly inhibited hepatic SOD activity, likely due to reactive oxygen species (ROS) overproduction [38]. Hepatic CAT activity initially increased, which may indicate a compensatory pattern associated with H_2_O_2_ accumulation; however, high-dose exposure suppressed CAT, indicating enzyme inactivation under oxidative overload. This suppression was exacerbated by DBP co-exposure, consistent with reports that phthalates impair ROS clearance by promoting ROS generation or inhibiting antioxidant enzyme function [39]. In the intestine, PSNPs exposure reduced SOD activity while CAT activity tended to increase, indicating tissue-specific oxidative stress induction. Although pollutant exposure typically activates antioxidant defenses as a protective mechanism [40], SOD suppression here suggests antioxidant system dysfunction. Following DBP co-exposure, intestinal SOD expression increased but CAT activity was suppressed, indicating incomplete compensatory adaptation. Elevated CAT activity normally alleviates oxidative damage by eliminating excess H_2_O_2_ [41]. However, CAT inhibition can occur when H_2_O_2_ accumulation exceeds clearance capacity, exacerbating cellular injury, consistent with DBP-induced responses in *D. rerio* [42]. Notably, *SOD* mRNA was up-regulated in both liver and intestine despite suppressed enzymatic activity, revealing a decoupling between transcriptional and enzymatic responses. Such discrepancies between transcriptional and catalytic responses have been documented in other species. For example, *Anguilla anguilla* exposed to contaminated sediments showed divergent trends in SOD gene expression and enzyme activity [43]. However, *O. niloticus* larvae exposed to microcystin-LR (MC-LR) exhibited no correlation between SOD transcriptional levels and enzymatic activity [44]. Studies on fish exposed to heavy metals (e.g., lead, cadmium) further indicate that transcriptional regulation of antioxidant enzymes does not consistently align with their functional antioxidant roles [45,46]. These inconsistencies suggest potential compensatory mechanisms between transcriptional regulation and enzymatic activity [47,48]. In these compensatory conditions, ROS are thought to be associated with reduced enzyme activity, potentially through direct or indirect protein impairment [49]. Additionally, divergent trends between gene expression and enzyme activity could arise from factors such as RNA stability, post-translational modifications, or temporal delays between transcription and protein synthesis [50]. Future work will incorporate time-series designs and proteomic analyses to explore these biological mechanisms.

MDA, a key lipid peroxidation (LPO) biomarker, significantly increased in the intestine under PSNPs+DBP co-exposure compared to PSNPs alone, indicating DBP compromises intestinal antioxidant capacity. However, neither PSNPs alone nor PSNPs+DBP co-exposure altered hepatic MDA levels. This aligns with studies showing no significant hepatic LPO/MDA changes in *O. niloticus* exposed to PSMPs [51] or *Pomatoschistus microps* exposed to polyethylene microplastics (PEMPs) [52,53]. Collectively, these findings suggest aquatic organisms may activate antioxidant defenses to mitigate microplastic-driven oxidative stress. Given the complex and sometimes paradoxical biological responses to MPs, influenced by particle characteristics and exposure conditions, further investigation into chronic biochemical responses in freshwater fish is critical for comprehensive ecological risk assessment.

Inflammation is a key physiological response to environmental stress in fish. In this study, low-dose PSNPs exposure significantly up-regulated hepatic mRNA levels of *IL-8*, *IL-1β*, *IL-10*, *TOR*, *IκBα*, and *NF-κB*, indicating immune activation under environmentally relevant conditions. This immune modulation is consistent with low-dose MP-induced cytokine activation in *Oryzias melastigma* [54]. In contrast, intestinal expression of inflammatory gene (*IL-1β, TOR, IκBα, NF-κB*) was suppressed with increasing PSNP concentrations. As an inhibitor of NF-κB, the up-regulation of *IκBα* suggests negative feedback regulation that modulates inflammatory responses during excessive NF-κB activation [55]. At higher PSNPs concentrations, enhanced *IκBα* expression likely inhibited *NF-κB* activation, dampening downstream inflammatory responses. Co-exposure with DBP led to dose-dependent up-regulation of *IκBα* and *NF-κB* in both liver and intestine, suggesting synergistic immune activation potentially mediated by ROS signaling. This aligns with the pro-inflammatory enhancement observed under phthalate exposure [56], highlighting the role of plasticizer co-contaminants. Exposure to high-dose PSNPs alone or combined with high-dose DBP significantly downregulated mRNA levels of key female reproductive genes, including *Bmp15, Cyp19a1a, Figla* and *Foxl2*, suggesting PSNPs or DBP at certain concentrations can impair ovarian development. This disruption of ovarian gene networks critical for oogenesis (*Cyp19a1a* [42], *Foxl2* [35]) aligns with reports of HPG axis vulnerability to diverse pollutants, including MPs and NPs. Such findings imply that environmental pollutants, particularly endocrine-disrupting chemicals (EDCs) like DBP, may disrupt reproduction by interfering with hormone synthesis and endocrine regulation, consistent with diethylstilbestrol-induced oogenesis disruption via HPG axis dysfunction [57]. The interpretation of the mRNA results is limited by the relatively small sample size and the lack of multiple testing corrections, which may have reduced the statistical power of the analyses. Future studies should incorporate larger sample sizes and apply appropriate multiple comparison procedures, such as the Benjamini–Hochberg correction, to mitigate the risk of false-positive findings and improve the conclusiveness of the results.

Given the established link between gut microbiota dysbiosis and metabolic disorders [58], we examined microbial shifts and their correlations with oxidative stress and inflammation to assess systemic toxicity. In terms of α-diversity, PSNPs exposure reduced ACE and Chao1 indices, whereas co-exposure slightly increased them; however, neither change was statistically significant. Similarly, Shannon and Simpson indices remained stable, indicating that the exposures did not markedly impair overall community diversity. In contrast, β-diversity analysis revealed pronounced structural differentiation, with PERMANOVA and ANOSIM showing that treatment factors explained approximately 35% of the variation. The PSNPs-High group exhibited distinct clustering, while the PSNPs-Low and some co-exposure groups displayed greater intra-group variability. These findings suggest that exposure dose and chemical co-exposure may affect the stability of gut microbial communities to some extent. Observed OTU richness declined under PSNPs but peaked in the PSNPs+DBP-Low group, possibly reflecting a compensatory hormetic response [59]. Notably, Proteobacteria, a dysbiosis and inflammation marker [60], was consistently reduced, potentially indicating microbial stress compensation [61]. Correlation analysis revealed a negative association between Proteobacteria abundance and SOD activity in hybrid snakehead. As persistent Proteobacteria enrichment typically indicates dysbiosis [60], its reduction here may relate to altered antioxidant capacity. At the genus level, *Acidobacterioter*, typically low-abundance gut anaerobes, showed negative correlation with MDA, suggesting potential mitigation of lipid peroxidation through anaerobic metabolism or other biochemical pathways. These alterations in the microbiome may contribute to the host health effects associated with microplastic exposure. However, due to the low replication in the microbiome analyses (n = 3), further studies incorporating larger sample sizes and functional profiling (e.g., metagenomics) are warranted to validate the observed microbiome–host interactions and elucidate their biological significance.

## 5. Conclusions

This study systematically investigated the individual and combined ecotoxicological effects of PSNPs and DBP on hybrid snakehead, simulating environmentally relevant exposure levels. PSNP exposure alone induced histopathological damage, oxidative stress, and inflammatory responses in the liver and intestine, accompanied by intestinal microbiota dysbiosis. Co-exposure with DBP significantly exacerbated these effects, highlighting the compounded ecological risks from the interaction of microplastics and co-contaminants, particularly in aquaculture systems. However, this study is limited by sample size and can only provide preliminary evidence. Future studies should employ a full-factorial design, more comprehensive histological analyses, broader dose gradients and time-series sampling, as well as proteomics and functional metagenomic analyses, to further validate these preliminary findings and elucidate the underlying mechanisms.

## Figures and Tables

**Table 1:** Growth parameters of hybrid snakehead following PSNPs alone exposure.

Group	IBW	FBW	WGR	SGR	SR
PSNPs-Ctrl	12.41 ± 0.71	23.63 ± 1.07 ^c^	90.42 ± 8.06 ^c^	2.94 ± 0.21 ^c^	76.00 ± 0.09
PSNPs-Low	12.80 ± 0.86	24.54 ± 0.92 ^c^	91.74 ± 7.21 ^d^	3.00 ± 0.17 ^c^	77.3 ± 0.08
PSNPs-Medium	12.50 ± 0.60	20.77 ± 0.79 ^b^	66.18 ± 5.54 ^b^	2.34 ± 0.16 ^b^	76.00 ± 0.09
PSNPs-High	12.20 ± 0.42	17.79 ± 0.80 ^a^	45.82 ± 6.53 ^a^	1.69 ± 0.17 ^a^	74.67 ± 0.08

Note: Significant differences are indicated by different letters in the same column (*p* < 0.05).

**Table 2:** Morphological parameters of hybrid snakehead intestine.

Group	Villus Length	Muscle Layer Thickness	Villus Count	Injury Rate 1	Injury Rate 2
PSNPs-Ctrl	195.86 ± 21.87	53.84 ± 9.79	20.0 ± 3.06	/	/
PSNPs-Low	192.88 ± 11.65	56.42 ± 13.05	23.0 ± 3.51	17.10 ± 1.45	/
PSNPs-Medium	186.50 ± 12.26	56.36 ± 18.92	23.0 ± 4.34	/	21.29 ± 7.90
PSNPs-High	175.67 ± 15.72	53.50 ± 10.30	24.0 ± 4.83	/	29.04 ± 14.89

Note: Damage rate 1 refers to the percentage of intestinal villi that are damaged after exposure to PSNPs alone relative to the total number of villi. Damage rate 2 refers to the percentage of intestinal villi that are damaged after combined exposure to PSNPs and DBP relative to the total number of villi.

## Data Availability

The corresponding data of hybrid snakehead can be accessed with accession No. PRJNA1312961.

## Supplementary Materials

The following supporting information can be downloaded at: https://www.mdpi.com/article/10.3390/antiox14091084/s1, Supplementary Table S1: The primers involved in qPCR; Figure S1: The rarefaction curves of groups and individual samples.
