# Supplementary material for: Single and Combined Effects of Polystyrene Nanoplastics and Dibutyl Phthalate on Hybrid Snakehead (Channa maculata ♀ × Channa argus ♂)
Source: Antioxidants (Basel). 2025 Sep 3;14(9):1084. doi: 10.3390/antiox14091084 (PMC12466840; doi:10.3390/antiox14091084)
Supplement: Supplementary file 1 [file antioxidants-14-01084-s001.zip › antioxidants-3736805-supplementary.pdf]

Supplementary Table S1. The primers involved in qRT-PCR.

**Type**	**Gene name**	**Primer name**	**Sequences (5'-3')**
	β-actin-F	β-actin-F	GCAAGCAGGAGTATGATGAG
	β-actin-R	β-actin-R	TTGGGATTGTTTCAGTCAGT
	GHR	GHR-DL-F	ACAGGAACCACACAGCCATT
		GHR-DL-R	GCATCCAGCCCATTTCAAC
	IGF1-1	IGF1-1-DL-F	CGCTCTTTCCTCTCAGTGGC
		IGF1-1-DL-R	CAATGGCTGAGCCCAATGAC
Growth-related gene	*IGF1-2*	IGF1-2-DL-F	GTTTGTGTGTGGAGACAGAGGC
		IGF1-2-DL-R	GCACGCACAGAGTGAGTTGG
	IGF2	IGF2-DL-F	GTCTTCGTCCAGTCGTTCGC
		IGF2-DL-R	TGTTGCCCCTGCTGGTTG
	GH	GH-DL-F	CCGATTACATTATCAGCCCTATTG
		GH-DL-R	AGTAGTTTTCATACGGAGCGAGC
	IL-8	IL-8-DL-F	GAGTCTGAGCAGCCTGGGAGT
		IL-8-DL-R	CTGTTCGCCGGTTTTCAGTG
	IL-1β	IL-1β-DL-F	GACACGATGCGATTCCTATTCT
		IL-1β-DL-R	CACTGGGCAGTCTTCTCGGA
	IL-10	IL-10-DL-F	TGGCAGTGAAGAAGACAT
		IL-10-DL-R	CTTTGAAGTGCTCAGGGA
Inflammation-related gene	*TOR*	TOR-DL-F	GAGCCTCTCTCATCCTCACCAC
		TOR-DL-R	GATTCATTCCTTTCTCTTTAGCCA
	IκBα	IκBα-DL-F	AAAATGTTACCGTGCCAGGAC
		IκBα-DL-R	ATGTATCACCGTCGTCAGTC
	NF-κB	NF-kB-DL-F	CAGCCAAAACCAAGAGGGAT
		NF-kB-DL-R	TCGGCTTCGTAGTAGCCATG
	SOD	SOD-DL-F	GCAGGACCCCACTACAAT
		SOD-DL-R	CTGAGCGATGCCTATGAC
	Bmp15	Bmp15-DL-F	ATCAAAGCCTCATTCGTCCA
		Bmp15-DL-R	GGCAGTGAGGGTCAAGTGTG
	Ctnd1	Ctnd1-DL-F	CGCTACCTGTCATCTCTGTGGA
		Ctnd1-DL-R	GGAGGTCCGTAGTGGTAGTTGC
	Ctnd2	Ctnd2-DL-F	ACATCGGAGCGGAAAGTTAC
		Ctnd2-DL-R	TGCCCTCTAAGGTTGGGTTTG
Gonadal-related gene	*Cyp19a1a*	Cyp19a1a-DL-F	AATACCCCTCGTCGTTACTT
		Cyp19a1a-DL-R	AAACCCTTATGGAGGCAAA
	Er	Er-DL-F	AGGTCCCGCGTCCTCTGTAT
		Er-DL-R	CCTGTTGAACCCGTGAATGTG
	Figla	Figla-DL-F	CAACGCCAAGGAACGACTG
		Figla-DL-R	CTCTCCATAGGTCATTGCCCA
	Foxl2	Foxl2-DL-F	GAGAAAAATAAAAAAGGTTGGCA
		Foxl2-DL-R	CGGCGTCTCCTGTAGTTCC

Pax4	Pax4-DL-F	CAACGGGTGCGTCAGTAAGA
	Pax4-DL-R	TAGACGACACGCTGGGCAC
Sox11b	Sox11b-DL-F	TTGGGCCGGTACTTGTAGTC
	Sox11b-DL-R	AATGCTCAAAGACGCGGAGA
Sox3	Sox3-DL-F	ATGCTTATCATATCCCTCAGGTCC
	Sox3-DL-R	TGAACGCGGCTTCCACATAC
Amh	Amh-DL-F	ATAGAGAGGCTGGGGAGACTCAA
	Amh-DL-R	TCTCACTGGATTGTTGGGGTCT
Amhr2	Amhr2-DL-F	ACAATGCCGCTTCAAATAAA
	Amhr2-DL-R	CAATGGCTGAGCCCAATGAC
Dmrt1	Dmrt1-DL-F	TCCCTGACCCCGTCCAAAG
	Dmrt1-DL-R	TCGCTGCCTCTCGGCTATC
Star	Star-DL-F	GAACAGGCTGGCAGGTCC
	Star-DL-R	CAATGGTCCAGCCGTCCT
Sox11a	Sox11a-DL-F	GGAGACGGTGCTGATGATTT
	Sox11a-DL-R	AAGAGGTGGAAGATGCTCAA

Supplementary Figure S1 The rarefaction curves of groups and individual samples.

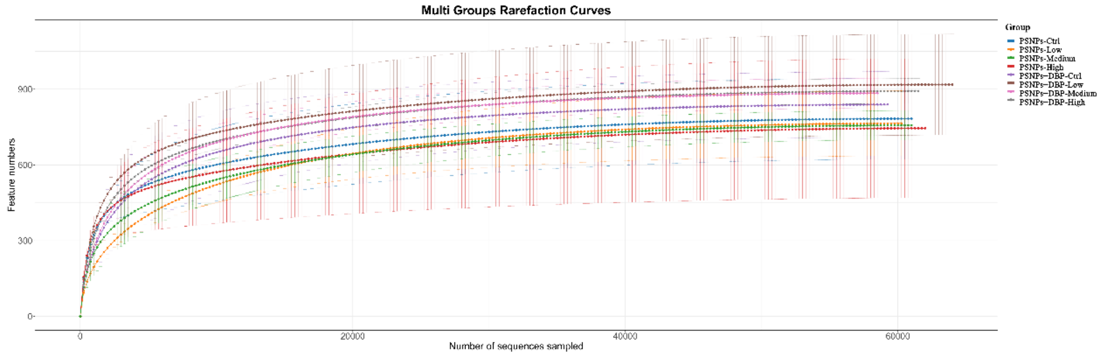

(A)

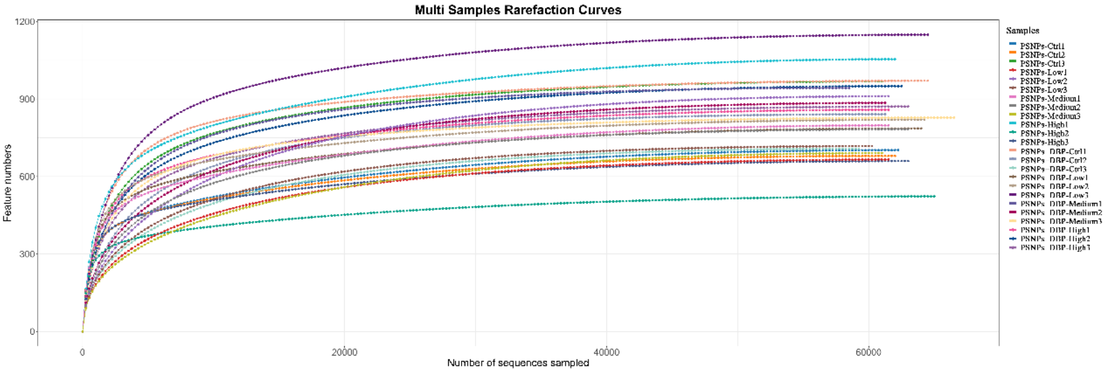

(B)

Figure S1. (A) Rarefaction curves of each treatment group showing species accumulation under different sequencing depths. (B) Rarefaction curves of individual samples reflecting microbial diversity at the single-sample level.
